# Spontaneous Acute Left‐Sided Abdominal Pain in a Patient With AL Amyloidosis

**DOI:** 10.1002/jha2.70350

**Published:** 2026-07-13

**Authors:** Shahzil Abdur Rehman Malik, Utkarsh Goel, Jason Valent, Faiz Anwer

**Affiliations:** ^1^ Medical College Aga Khan University Karachi Pakistan; ^2^ Department of Internal Medicine Cleveland Clinic Cleveland Ohio USA; ^3^ Department of Hematology and Medical Oncology Taussig Cancer Center Amyloidosis Center, Plasma cell Disorders Program Cleveland Clinic Cleveland Ohio USA

**Keywords:** AL amyloidosis, atraumatic abdominal pain, hemoperitoneum, splenic hemorrhage, splenic rupture

1

A 69‐year‐old female with IgG lambda AL amyloidosis involving the heart and skeletal muscle, diagnosed six months earlier following a left biceps muscle biopsy performed for evaluation of progressive muscle weakness, presented to the emergency department with four days of progressive, sharp left‐sided abdominal pain, exacerbated by movement and lying flat. She was treated with daratumumab, bortezomib, cyclophosphamide, and dexamethasone beginning in January 2025 and achieved a hematologic complete response by February 2025, one month prior to her presentation in the emergency department. The pain was associated with nausea, vomiting, and decreased appetite. She denied fever, diarrhea, or recent trauma. Physical examination revealed stable vital signs and localized left upper quadrant tenderness without guarding or a palpable mass.

Computed tomography of the abdomen demonstrated splenomegaly (maximum diameter 13.9 cm) with subcapsular and pericapsular hemorrhage, a sentinel clot, and small‐volume hemoperitoneum. These findings were consistent with spontaneous splenic rupture with bleeding (Figure 1), a rare but serious complication of AL amyloidosis. Surgical intervention was initially deferred; however, persistent pain and concern for recurrent bleeding prompted a multidisciplinary discussion between the surgical and oncology teams. Following vaccination against encapsulated organisms, a semi‐urgent laparoscopic splenectomy was performed three days later. The removed spleen weighed 236.3 g, and its dimensions were 17.5 cm × 12.6 cm × 3.8 cm. The identifiable capsule was purple‐gray and smooth. The cut surface of the spleen was dark red and firm.

**FIGURE 1:**
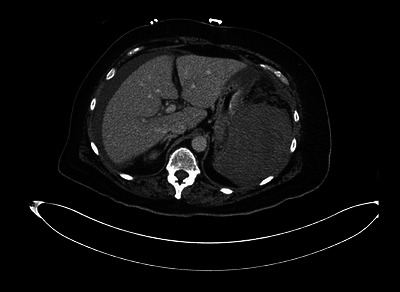
CT abdomen showing irregular, enlarged splenic appearance with evidence of subcapsular and pericapsular sentinel clot. Small volume abdominopelvic hemoperitoneum is also seen.

Postoperative biopsy demonstrated extensive Congo red‐positive amyloid deposition replacing nearly all normal splenic architecture. Only focal areas with residual splenic tissue were identified with no morphologic evidence of involvement by a plasma cell neoplasm or lymphoproliferative disorder (Figure 2). The patient tolerated surgery well and was discharged home in a stable condition on postoperative Day 4.

**FIGURE 2:**
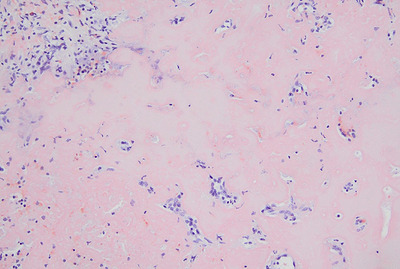
Extensive Congo red positive amyloid deposition replacing nearly all of the normal splenic architecture.

AL amyloidosis predisposes patients to bleeding due to extracellular amyloid deposition within vascular and parenchymal tissues, resulting in increased tissue fragility [1]. While cutaneous manifestations such as easy bruising and periorbital ecchymosis are more common, spontaneous splenic rupture is a rare but potentially life‐threatening complication documented in a few case reports. Early recognition is critical, as delayed diagnosis may lead to catastrophic intra‐abdominal hemorrhage [2]. In the absence of evidence‐based management guidelines, expert opinion supports early vaccinations against encapsulated organisms followed by splenectomy in patients with spontaneous splenic rupture with subcapsular and pericapsular hemorrhage due to high risk of recurrent bleeding [3].

## Author Contributions

FA and JV conceptualized the study. SARM and UG contributed to the initial draft of the manuscript. SARM, UG, JV and FA contributed to the reviewing and editing of the manuscript. All authors reviewed the final draft, and suggested changes in the manuscript and approved the final manuscript.

## Funding

The authors have nothing to report.

## Consent

Written informed patient consent has been obtained for publication of the case details and associated images.

## Conflicts of Interest

FA: Consulting or Advisory Role: BMS, Poseida Therapeutics, GI Innovation, Caribou Biosciences Speakers' Bureau: Bristol Myers Squibb Foundation Research Funding: Celgene (Inst), Bristol Myers Squibb (Inst), Caribou Biosciences (Inst), Caribou Biosciences Travel, Accommodations, Expenses: Bristol Myers Squibb Open Payments Link: https://openpaymentsdata.cms.gov/physician/16726. JV: Alexion, AstraZeneca Rare Disease (Research Funding). No other conflicts of interest to disclose.

## Data Availability

The data that support the findings of this study are available from the corresponding author upon reasonable request.
